# Experiments and Observations on the Atmosphere of Marshes

**Published:** 1802-02

**Authors:** Adam Seybert


### *Experiments and Obſervations on the Atmoſphere of Marſhes.*

*By* Adam Seybert, M. D.

WHEN inquiries which have attracted the attention of a Franklin, a Prieſtly, an Ingenhousz, and many other eminent perſons, without being decided, are undertaken by one whoſe abilities are ſo far inferior as mine, little ſucceſs can be expected. This remark announces the difficulty of the ſubject I am about to inveſtigate; nevertheleſs, I am ſtimulated by the induſtry of my predeceſſors, and if I cannot promiſe much new matter, I hope to be at leaſt able to verify ſome obſervations and perhaps diſprove others; for in proportion as we remove errors we approach nearer to truth.

When we are fully perſuaded, that to live and to breathe are ſynonimous terms, and that the abſolute neceſſity of air to the maintenance of animal life has been fully eſtabliſhed by repeated and well concerted experiments, we need not be ſurpriſed to find many perſons engaged in an examination of the chemical qualities of our atmoſphere: the names of Scheele, Prieſtly, Lavoiſier, Fontana, &c. will for ever make this branch of ſcience reſpectable.

From the earlieſt ages it has been ſuppoſed that the atmoſphere has great influence on the human body in producing diſeaſe, as well as in reſtoring health; hence the accounts of Hippocrates, Sydenham and Huxham. Phyſicians ought always to notice the ſtate of the atmoſphere during the prevalence of epidemic diſeaſes.

Before facts were collected and experiments well performed, the atmoſphere was ſuſpected to differ materally in almoſt every ſituation; but latter experiments have proved that our notions have been erroneous to a great degree.

In a former memoir which I had the honour to read before your ſociety, I paid particular attention to the atmoſphere over the ocean, rivers and neighbouring land, and hope that my experiments have been of ſome ſervice towards the eſtabliſhment of truth; in the preſent eſſay I intend giving an account of ſome experiments which I peformed at different times on the air over marſhes.

A few general remarks reſpecting the common ſtate of our atmoſphere, perhaps become neceſſary, for the ſake of future compariſons.

We no longer believe, for experiments have taught us the contrary, that our atmoſphere is an homogeneous element; the preſent ingenious doctrines of heat have thrown much light upon the ſubject; and with much reaſon ſome philoſophers are induced duced to believe "that the aeriform ftate is a modification of bodies, dependent on the degree of temperature, and on the preffure which thefe bodies undergo!"* This opinion has been extended fo far as to induce fome to fay, "Perhaps alfo metals are contained in the atmofphere."† Thefe fentiments do not appear to be merely conjectural, for Chaptal has precipitated mercury from oxygen gas (which was obtained from red precipitate) by means of ice; and the family of Achard, fuffered ptyalifm from breathing in an atmofphere where mercury had been expofed for fome time in a faucer.

The above opinions, if true, (and I think them highly probable) prepare us to meet many difficulties in the analyfis of the atmofphere. But all I expect to do is to open the paffage, and I fhall leave others to render it more certain; for numerous experiments, and thofe often repeated, are the only means whereby we can afcertain truth; and I fear the labours of one man are infufficient to perform this tafk.

I am not without hopes that others will engage in the inquiry, it is of importance to every citizen, more efpecially fince we find that our principal cities are almoft yearly afflicted with a terrible epidemic, which has been by fome attributed to the ftate of the air. Future difcoveries, it is hoped, will multiply the number of tefts for airs, and thus render the fubject more eafy.

Refpectable chemifts have determined the component part of our common atmofphere to be

| | | |
|---|---|---|
| Oxygen gas | - - - | 27. |
| Azotic gas | - - - | 72. |
| Carbonic acid gas | - | 01. |

Any deviation from this ftatement muft be attributed to local circumftances.

I fhall firft endeavour to determine, whether or not the air of marfhes differs from that of other fituations:

2. What are the caufes of the differences which are found to exift:. And,

3. Make a few obfervations and remarks.

1. March 31ft, 1798. Air was obtained by agitating ftagnant water over marfhy grounds; the following experiments were then performed.

*a.* It burned when a candle was applied to it; the flame was blue: it did not explode when mixed with atmofpheric air.

*b.* Agitated,

* Lavoifier's Elements of Chemiftry, p. 59.

† Girtanner's Antiphlogiftiche Chemie, p. 58.

*b.* Agitated with lime water, a copious white precipitate was formed.

*c.* Its bulk was confiderably diminifhed by agitating it with lime water.

*d.* Equal bulks of it and nitrous gas were introduced into my eudiometer tube, and a diminution of $\frac{2}{100}$ of their bulk was perceptible.

Thefe experiments were frequently repeated, and the refults were fimilar to the above-mentioned: they warrant the following inferences:

*a.* That carbonic acid gas enters largely into the compofition of the air examined.

*b.* That hydrogen gas is an ingredient in it.

*c.* That no oxygen gas was prefent: for the fmall abforption which took place, I attribute to the action of the water with which the airs were agitated.

The above experiments were performed on the air, which was obtained immediately as it was difengaged from the marfhy foil; it became neceffary to examine the air fituated at fome diftance above the marfh.

At different times during the fummer of 1798, I collected air from above marfhy grounds: the following experiments were performed on it.

*a.* When agitated with lime water, it afforded a precipitate, which was not fo abundant as in the former experiments.

*b.* Mixed with nitrous air, its bulk was diminifhed to almoft as great a degree as the air in the yard of my lodgings.

*c.* Either pure or mixed with atmofpheric air, it did not burn or explode when a candle was applied to it.

Hence it appears that the air obtained at the height of feveral feet above marfhes,

1. Contains little or no hydrogen gas.
2. That the proportion of carbonic acid gas is pretty confiderable; and,
3. What is of great confequence to be obferved, a large quantity of oxygen gas enters into its compofition.

The laft mentioned facts induce us to believe, that the air *above* marfhes is not confiderably different in its properties from the common atmofphere in other fituations, where animals refpire with eafe and enjoy perfect health, except the proportion of carbonic acid gas being greater; and this I am induced to believe diminifhes in quantity as we afcend: for facts related by travellers who have vifited the Grotto del Cane and other fimilar places, prove that the gravity of this elaftic fluid permits it to rife but to an inconfiderable height.

After having proved that certain qualities do exift in the air over marfhes, which are different from thofe poffeffed by the atmofphere in other fituations, we muft next attend to our

Second object, viz. to afcertain what are the circumftances about marfhes which produce fuch effects ?

Before we proceed any further, it is of the greateft importance to be fatisfied refpecting the changes which may be produced on common atmofpheric air, when fubjected to the action of the foil of marfhes.

At different times in the months of September and the commencement of October 1796, I expofed atmofpheric air to the action of mud, which I obtained from marfhes below the city. The fame was done at different times in the months of April and July, 1798. The experiments were expofed to the temperature of the atmofphere. The refults from the different experiments were fimilar. The air was expofed to the action of the mud which was contained in a tumbler, by means of an inverted glafs jar, in a bafon containing a fmall quantity of water. The following changes were noticed.

1. The air contained in the jar became much diminifhed in bulk, as was proved by the water rifing into the jar.

2. The air, thus acted upon, when agitated with lime water, afforded a copious white precipitate and became diminifhed in bulk.

3. In fome of the jars, were fufpended papers, ftained blue with litmus and yellow with turmeric; the blue received a reddifh tinge and the yellow remained unaltered; the red was again changed to a blue by expofure to the vapour iffuing from a bottle containing ammoniac.

4. The air thus altered by the mud, when mixed with nitrous gas in the eudiometer tube, was in every inftance found to have loft in point of purity; fometimes no diminution of bulk whatever took place.

The following circumftances feemed to influence the laft mentioned experiments. 1ft. Temperature. 2d. The length of time during which they were continued. And 3d. The proportion which the mud and air bore to each other; the furface of the mud being more or lefs extenfive, feemed alfo to have its effects.

The air thus affected by the action of the mud would in no inftance burn or explode, when a candle was applied to it; hence it contained but a fmall quantity of hydrogen gas.

This laft mentioned fact induced me to engage in an effay to determine the origin of the hydrogen gas which abounds in the *air obtained by agitant ftagnant waters.*

It is neceffary to be obferved, that in the above experiments with mud, but a fmall proportion of water was added to it in the the tumbler, the quantity was juſt ſufficient to promote putrefaction. I am of opinion that the hydrogen gas is afforded by a decompoſition of the ſtagnant water, effected by the putrefaction of the dead animal and vegetable ſubſtances, which enter largely into the compoſition of the ſoil of marſhes. I was induced to form this opinion, becauſe, firſt, pure water is a compound of but two elements, conſequently the affinity cannot be broken but by the action of a third ſubſtance. And ſecondly, we have no experiments which prove that pure water has undergone ſpontaneous decompoſition. My ideas are confirmed by a fact well known to all ſeamen, viz. when a candle is applied to the bung hole of a caſk containing *river water*, which had been for ſome time cloſely ſtopped, an elaſtic fluid eſcapes, which will inflame, and appears in all reſpects ſimilar to hydrogen gas obtained by other means.

After forming the above conjectures, I determined to perform a few experiments which might tend to confirm or diſprove my opinion. With this view, mud and water, with a very ſmall portion of atmoſpheric air, were at different times confined in bottles cloſely ſtopped and inverted over water: in ſome inſtances the experiment was continued during 20 and 30 days. They were ſubjected to the temperature of the atmoſphere. During the progreſs of the experiments, I perceived that an elaſtic fluid was diſengaged from the materials contained in the bottles, and that the water was evidently diminiſhed in bulk. The elaſtic fluid generated during theſe experiments, 1ſt, inſtantly formed a copious white precipitate when agitated with lime water; 2dly, it burned, when the flame of a candle was applied to it, and poſſeſſed the other properties which are common to air obtained by agitating ſtagnant waters over marſhes.

Theſe facts are deciſive to me on the ſubject, and confirm the above conjectures reſpecting the origin of the hydrogen gas diſengaged from marſhy grounds. It is neceſſary to remark, that ſome danger attends theſe laſt experiments; for a large bottle which was cloſed by a ground ſtopper, was broken on the 25th day of the experiment, by an expanſion of the contained elaſtic fluid: the pieces, which were large, were thrown to the diſtance of 20 feet, and a report was heard louder than that from the firing of a muſket. In general, the bottles had corks faſtened by means of ſtrings bound round them: as ſoon as I cut the ſtrings, the corks were forced from the necks of the bottles with conſiderable violence.

The above experiments teach us that mud vitiates the atmoſphere in a very powerful manner. They alſo enable us to account for the preſence of the elaſtic fluid forming the atmoſphere of of marſhes. It appears, that the carbone of the mud unites with the oxygen of the decompoſed water, and forms the carbonic acid gas, whilſt the hydrogen gas is ſet at liberty. Theſe are truths not to be invalidated by gratuitous aſſertions, ſince their baſis is experiment.

It may be aſked, if mud ſeizes oxygen gas with the avidity ſtated, how comes it that eudiometrical experiments prove the air over marſhes to be nearly, if not quite, of the ſame degree of purity as that of other ſituations?

At firſt an anſwer to this important queſtion may ſeem difficult; but ſome examination of the circumſtances attending the ſituation of marſhes, enables us to account for it in a very ſatisfactory manner. It is to be remarked that in my trials with mud, the air was confined under glaſs veſſels over water, conſequently no circumſtances from without could have any influence on the experiments. The air over marſhy ſituations is very different, it poſſeſſes all the advantages of ventilation, &c. in common with the atmoſphere. Beſides theſe circumſtances, a large quantity of oxygen gas is afforded by the living vegetables which ſurround them in abundance. We may alſo obſerve, that frequently large ponds of water are found in their neighbourhood, and that often rivers are at no great diſtance from them: may not therefore a quantity of oxygen gas be diſengaged from theſe waters by the action of the ſun? Experiments are related by reputable authors, wherein water has been decompoſed by the action of the ſun's rays; of this more hereafter.

That the atmoſphere of marſhes, therefore, differs in certain circumſtances from that of other ſituations, and that the ſoil has conſiderable effect in altering the air of the atmoſphere, I think, cannot be doubted. Let us therefore endeavour to diſcover the particular local cauſes which give riſe to theſe variations.

I have before hinted that the putrefaction of the animal and vegetable matters upon the ſoil of marſhes, was the great cauſe of the changes obſerved to exiſt; for every ſpecies of ſoil will not operate in the manner alluded to.

That the cauſe is in the putrefaction of theſe matters, and that this ſtate is abſolutely neceſſary to thoſe changes, I infer from the following circumſtance; marſhes have no noxious influence during the winter ſeaſon. They cauſe diſeaſe when the circumſtances are preſent which promote putrefaction; as a proper degree of heat, a due quantity of moiſture, and the contact of atmoſpheric air or ſubſtances capable of affording oxygen, as *water*. That a certain degree of moiſture is neceſſary, appears evident from White's experiments, related in the Philoſophical

Tranſactions:

Tranfactions: he fays, " a certain degree of moifture feems neceffary to produce the bad effects of marfhes; for mud when perfectly dry did not alter the air." He might have added, that too much fluidity will likewife prevent their bad confequences, which is proved by the neighbourhood being healthy when they are overflowed. An overflow of water may operate by preventing the powerful effects of the fun. Experience teaches us, that their bad effects are difcontinued when they become dry. Covering them with clay and other fubftances not liable to putrefaction, deftroys their bad effects, fo does cultivation, froft, &c.

Living trees being planted in their neighbourhood renders the fituation more healthy, by abforbing the gas exhaled during putrefaction and affording oxygen gas.

White's experiments prove, " 1ft. During fixteen hours, air confined in a phial over water did not fuffer a change. 2dly. Pure clay moiftened did not alter the purity of the air. 3dly. Sand moiftened did not change the purity of the air. But 4th. *Mud (which confifts of earths intimately mixed with dead animal and vegetable fubftances) rendered the air very impure, as I proved by the experiments which I performed.*

The following reflections occurred to me fome time fince, and are copied from my note book.

To arrive at any certain knowledge refpecting the manner by which marfhes can be fuppofed to affect the atmofphere, we muft inveftigate their compofition.

They feem to confift of,

1ft. More or lefs water. 2dly. Different proportions of *dead* animal and vegetable matters. And 3dly, The earthy fubftances compofing the original foil.

Animals and vegetables, when they have fuffered death, are fubject to the laws which govern inanimate matters in general, and they are liable to the various changes produced by chemical mixture and the laws of chemical affinity: they are acted upon by the powerful agents of nature, and thus fuffer decompofition, and form new combinations.

All chymifts acknowledge the analyfis of animal and vegetable fubftances to be imperfect. Lavoifier has paid particular attention to the fubject. He performed numerous and accurate experiments to determine their compofition, and notices in a particular manner the refults they afford during their putrefaction. According to him, they confift chiefly of hydrogen and oxygen, combined with carbone: thefe fubftances, he fays, are found in all vegetables, and none exift without them. Animal fubftances contain more hydrogen and azote than vegetables do, they alfo have carbone as a conftituent part of their compofition: fome of both claffes contain fulphur and phofphorus.

The

The above are the principles which I ſuppoſe are liable to be acted upon, and thus produce the effects we are about to conſider.

Before we can underſtand the changes to which the above ſubſtances are liable, we muſt take into conſideration, that our atmoſphere is compoſed of the azotic and oxygen gaſes, and a ſmall portion of carbonic acid gas: many view this laſt as adventitious and by no means neceſſary.

Heat, moiſture, the contact of atmoſpheric air and reſt, we know are circumſtances attendant on marſhy ſituations during the unhealthy ſeaſons.

*A priori*, we might be induced to believe that the following phenomenon would take place, under the above circumſtances.

1. That hydrogen gas would be diſengaged. 2. That the oxygen combining with the carbone would form the carbonic acid gas. 3. That azote would unite with a portion of hydrogen and thus produce ammoniac; whilſt another portion of it would, during its combination with oxygen, form the nitric acid. And 4th. That when ſulphur or phoſphorus were preſent, they with hydrogen would form the ſulphurated and phoſphorated hydrogen gaſes.

We ſhall now endeavour to diſcover whether or not theſe elaſtic fluids enter into the compoſition of the atmoſphere of marſhes.

1. Hydrogen gas. Doctor Franklin has long ſince demonſtrated the production of this elaſtic fluid in marſhy ſituations. Ingenhouſz and others have confirmed the truth of his experiments and obſervations.

My experiments convince me that it is produced in a conſiderable quantity, and that it may be eaſily procured by agitating ſtagnant waters over marſhes. It is alſo evident that this gas is in a ſtate of mixture with the carbonic acid gas.

Although we are certain that a large quantity of hydrogen gas is diſengaged from marſhy grounds, we muſt nevertheleſs conclude that it bears but an inconſiderable proportion to the atmoſphere at large; for we find that the air immediately above marſhes will not explode upon the approach of a candle: indeed, from its levity, we might ſuppoſe that it occupies the inferior ſtrata of the atmoſphere but for a ſhort time.

2. Carbonic acid gas. That this elaſtic fluid enters largely into the compoſition of the atmoſphere of marſhes, is eaſily proved by agitating it with lime water.

3. Ammoniacal gas. The production of this gas during putrefaction is proved beyond doubt; therefore that it ſhould exiſt in the atmoſphere of marſhes ſeems at leaſt probable, indeed many have inferred conſiderable effects from its preſence; but as they they did not detect it by any teft with which we are acquainted, their opinion is entirely hypothetical.

The following are the refults of the means I employed to difcover whether ammoniacal gas is prefent in the atmofphere of marfhes. 1. No white clouds appeared, when muriatic acid gas was mixed with air obtained by agitating ftagnant waters. 2. Slips of paper ftained yellow by turmeric, were fufpended in a bottle containing mud and atmofpheric air, it remained unchanged; whereas thofe ftained with litmus received a reddifh tinge. 3. I never could perceive the odour peculiar to this alkali, when I vifited marfhes.

The above experiments caufed me to doubt the prefence of this elaftic fluid in the atmofphere of marfhes. I was confirmed in this opinion by the following circumftances: 1ft. Ammoniac combines readily with water: it is impoffible to procure ammoniacal gas over water; therefore we are to fuppofe that if this fluid is produced it is immediately abforbed by the water of the marfh. 2dly. Carbonic acid gas is abundant in the atmofphere of marfhes. By experiment, I afcertained that this acid and ammoniacal gas were very prone to unite and form the carbonat of ammoniac. The experiment was performed in a glafs tube over mercury: as foon as the two elaftic fluids came in contact, an abforption took place and the bulk of them was confiderably diminifhed: at the fame time the fides of the tube were encrufted with a white matter, which poffeffed all the properties of the carbonat of ammoniac. If fuch are the phenomena of thefe experiments, why will not fimilar effects take place in marfhy fituations?

4. Nitric acid. The experiments and obfervations of Thouvenel and others, have long fince demonftrated the production of this acid during putrefaction. If it is formed in marfhy fituations, its prefence cannot be proved in their atmofphere, and I am inclined to believe that it is immediately abforbed by the neighbouring waters.

5. Sulphurated and phofphorated hydrogen gafes. If thefe elaftic fluids confift of hydrogen gas, holding fulphur and phofphorus in folution, it feems probable that they fhould be generated during the putrefaction of fuch matters as contain them as conftituent elements. Although Chaptal in his Memoirs de Chimie, p. 141, obferves: "Que la boue noire, degagèe de tout vègetal, ne donnoit plus d'air inflammable mais rèpandoit une odeur de foie de foufre." Still he relates no experiment whereby he detected its prefence in the atmofphere of marfhes. Its ready abforption by water; marfh air when agitated with a folution of the acetite of lead producing no change in it; filver not tarnifhing fooner in thefe than in other moift fituations; and the air poffeffing no peculiar fmell, are all facts which tend to convince me that it does not exift; moreover Kirwan fays, that hepatic gas united with nitrous air will depofit fulphur. I agitated marfh air and nitrous air together in a glafs tube and no fuch phenomenon was noticed.

6. Azotic gas. If you burn candles in the air of marfhes, until all the oxygen be abforbed, and then agitate the remaining air with lime water, fo as to abforb the carbonic acid, an elaftic fluid ftill remains which poffeffes the properties of azotic gas.

7th and laftly. Oxygen gas. A variety of facts prove that oxygen gas is a principal ingredient in the atmofphere of marfhes. 1ft, Candles burn therein with the fame luftre as in other fituations. 2. Animals breathe with equal eafe as in other places. 3. Eudiometrical experiments prove that it forms as great a proportion here as in other atmofpheres which are reckoned more healthy.

Auguft 4th and 5th, 1796—July 8th and 10th, 1798—I collected air from over marfhy grounds to the fouth and north of Philadelphia; when tried with the Eudiometer, they always proved as pure as the air in the yard of my lodgings. Chaptal in his Memoirs de Chimie, p. 141, afferts that the air over the ponds, which border on the Mediterranean fea (the neighbourhood of which is equally marfhy if not more fo than the neck formed by the junction of Schuylkill and the Delaware, as I convinced myfelf during my refidence at Montpellier in the years 1795 and 1796) was equally pure with that of Montpellier, tried the fame day. When I affert that the atmofphere of marfhes is equally pure with that of other fituations, I mean that it contains as large a proportion of oxygen gas as fuch other atmofpheres do. I do not by any means intend to be underftood that it is free from foreign mixtures.

I have acknowledged that putrefaction is going on in marfhy places, and likewife admit that this procefs deftroys the purity of the atmofphere by abforbing its oxygen; therefore it may feem difficult to admit the *abfolute purity* of the air being equal here to that of other places. People being able to breathe with eafe over marfhy grounds, is fufficient proof that the oxygen gas there is adequate to fupport life. I fhall now attempt to account for the purity of the air of marfhes as follows. Sennebier has proved by numerous experiments, that living vegetables placed in an atmofphere of carbonic acid gas or in water faturated with this air, expofed to the action of the fun, thrive and grow very rapidly: during the experiments the carbonic acid is deftroyed and oxygen gas is difengaged. In addition to thefe experiments, Ingenhoufz has taught us that the aquatic plants, plants, particularly ſuch as grow in the neighbourhood of marſhes, poſſeſs the power above ſtated to a ſupriſing degree; ſee Experiences ſur les Vègètaux, Tom. 2. p. 401. Theſe facts, when properly conſidered and connected with the remarks I made when ſpeaking of the effects of mud on the atmoſphere, I think are ſufficient to account for the phenomenon, which at firſt ſeemed at leaſt doubtful.

The above view of this difficult ſubject will perhaps in ſome meaſure alter our opinions reſpecting the utility of marſhes. Heretofore mankind ſeem to have viewed their exiſtence as noxious to them, and unneceſſary to their happineſs. I confeſs my former opinion reſpecting them coincided perfectly with that of the majority, but at preſent my ideas are very different: I conſider them as very neceſſary to keep the atmoſphere in a *proper degree* of purity, for it is not only the impure atmoſphere which kills animals, but the too pure alſo; and an ingenious philoſopher has well obſerved, that animals live too faſt in atmoſpheres overcharged with oxygen gas. They appear to me to have been inſtituted by the Author of Nature, in order to operate againſt the powers which vegetables and other cauſes poſſeſs of purifying the atmoſphere, ſo that the oxygen may exiſt in a proper proportion, fit to ſupport animal life and combuſtion. I am of opinion, that ere long marſhes will be looked upon by mankind as gifts from Heaven to prolong the life and happineſs of the greateſt portion of the animal kingdom. Perhaps it was originally intended that they ſhould remain uninhabited, and that their only uſe ſhould be that of correcting the too pure atmoſpheres. Although their immediate inhabitants ſuffer diſeaſe from them, ſtill but a ſmall portion of the human race chooſe marſhy ſituations for their reſidence.

After I had read the above before the ſociety, a friend in converſation with me, objected to the operation of marſhes on the atmoſphere being intended to prevent a ſuperabundance of oxygen gas; he obſerved that this effect would be fully accompliſhed by the ordinary combuſtion and the reſpiration of animals. Upon reflection, his objections gave riſe to new confirmations of what I aſſerted: I remarked to him, that very extenſive tracts of country were ſufficiently warm without fires; that in theſe places nature gave uncommon powers to vegetable action, but at the ſame time ordained, that in theſe very ſituations marſhes ſhould be moſt abundant. If we view moſt ſouthern countries, I believe the above facts will be found to exiſt very generally. A further beautiful demonſtration of my propoſition may be adduced from a well known fact, that when vegetable life becomes paralized in the winter ſeaſon the operation of marſhes is then unneceſſary, and is likewiſe ſuſpended by the ſame cauſes, viz. froſt, &c.

